# Proteolytic stabilization of a spider venom peptide results in an orally active bioinsecticide

**DOI:** 10.1002/ps.8980

**Published:** 2025-06-18

**Authors:** Breck R. Davis, Alexandra M. Haase, Joseph S. Tourtois, Daniel L. Hulbert, Rachel E. Cornell, Brian T. DeVree, Cadence J. Flohrschutz, Lucille M. Bell, Daniel C. Peck, Trang T. Nguyen, Lin Bao, Robert M. Kennedy, Kyle D. Schneider

**Affiliations:** ^1^ Vestaron Crop Protection Kalamazoo Michigan USA; ^2^ Present address: Animerra, Inc. Boston Massachusetts USA; ^3^ Present address: Valent BioSciences Libertyville Illinois USA; ^4^ Present address: Fulbright Scholar Galápogos Ecuador; ^5^ Present address: Kalamazoo County Health and Community Services Kalamazoo Michigan USA

**Keywords:** peptides, crop protection, insecticide, sustainable agriculture

## Abstract

**BACKGROUND:**

The toxin peptide U1‐AGTX‐Ta1b from the Hobo spider, *Eratigena agrestis* (Walckenaer, 1802)*,* was studied to determine its potential to serve as a bioinsecticide.

**RESULTS:**

U1‐AGTX‐Ta1b has insecticidal potencies similar to commercial insecticides when injected directly into insect hemolymph but lacks activity when ingested by lepidopterans due to trypsin‐like gut proteases. Alanine scanning identified an arginine and lysine rich patch on the peptide's surface that is critical for bioactivity. Targeted stability studies on these basic residues identified a single site, R9, to be the rate limiting site in U1‐AGTX‐Ta1b proteolysis. Mutation of position R9 to glutamine was sufficient to stabilize the peptide and render the toxin orally active with the additional benefit of enhanced temperature stability. Further refinement of the peptide to remove an O‐linked glycosylation site and prevent exoprotease activity during expression in yeast led to a final peptide sequence suitable for commercialization as a bioinsecticide. This peptide displayed activity comparable to commercial insecticides in a range of crop/pest combinations.

**CONCLUSION:**

A novel, peptide‐based bioinsecticide derived from spider venom was developed to be stable and active by ingestion by lepidopteran pests. The peptide, U1‐AGTX‐Ta1b‐QA, can replace or reduce the use of chemical insecticides and has been approved by the United States Environmental Protection Agency. © 2025 The Author(s). *Pest Management Science* published by John Wiley & Sons Ltd on behalf of Society of Chemical Industry.

## Graphical abstract

Selective editing out of an insect gut protease cleavage site renders a spider‐derived insecticidal peptide active by ingestion by insect pests.

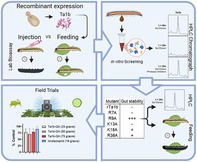

## 1 INTRODUCTION

According to the United Nations, 20–40% of the world's food crops are destroyed by insect pests each year.^1^ To manage insect pressure and maximize crop yields, growers must repeatedly apply insecticides over a growing season leading to the development of insecticide resistance.^2^ This forces growers to become reliant on fewer insecticide chemistries and to apply insecticides more frequently, increasing the rate of insecticide resistance evolution.^3^ Further, the most commonly applied insecticides are small‐molecules that can have problematic environmental and health profiles. Neonicotinoids, for example, have been reported to be a major driver of declines in pollinators and other non‐target arthoropods.^4^, ^5^ In response, world governments have tightened regulations around the use of many insecticides and increased scrutiny of the approval of new chemistries. To ensure a robust and affordable food supply, new insecticides with improved environmental profiles that avoid currently prevalent mechanisms of insecticide resistance are urgently needed.

Microbial and biological insecticides (bioinsecticides) are the main alternative to small‐molecule chemical insecticides and offer superior advantages. Unlike chemical insecticides, bioinsecticides are usually biodegradable and reduce environmental contamination. They often provide more specific target pest control than chemical insecticides and are a source of novel pest control tools to compensate for the shrinking chemical solutions as a result of pest resistance and governmental de‐registration. The bioinsecticide global market includes viruses, microbial isolates, plant extracts, pheromones, and minerals^6^ and was valued at $90.5 M in 2022.^7^ However, bioinsecticides represent less than 1% of the total ~$20B spent per year on insecticides, with 85% of the total market controlled by small‐molecules targeting ion channels and receptors in the peripheral and central nervous systems of insects.^8^ A bioinsecticide that combines the environmental and safety profile of biologicals with the nervous system activity of traditional insecticides represents a significant opportunity for development of new insecticides that are both safe and highly effective.

Certain arthropod predators have evolved venoms containing disulfide‐rich peptides that paralyze and kill their prey, sometimes by targeting the same nervous system receptors used by traditional insecticides. This indicates the peptides themselves could be a source of novel bioinsecticide leads.^9^, ^10^, ^11^, ^12^ Though potent, venom peptides have evolved to be directly injected into the insect hemolymph and so have not undergone selection pressure to withstand the insect digestive system. Additionally, commercial insecticides must conform to rigorous regulatory and field performance standards that require defined product homogeneity and stability, properties unlikely to have evolved in the native venom peptide.

In 2017, the United States Environmental Protection Agency (EPA) approved the first venom peptide insecticide, GS‐omega/kappa‐HxTx‐Hv1a (GS‐ω/κ). This compound was modified from an insecticidal peptide (ω/κ‐HxTx‐Hv1h) isolated from the Australian Blue Mountains funnel‐web spider (*Hadronyche versuta* (Rainbow, 1914)), demonstrating that venom peptides offer an alternative to small‐molecule insecticides. Here, we describe the engineering and development of the second EPA approved venom peptide insecticide, U1‐AGTX‐Ta1b‐QA, that was isolated from the venom of the hobo spider (*Eratigena agrestis* (Walckenaer, 1802)).

## 2 MATERIALS AND METHODS

### 2.1 Expression and purification of Ta1b variants

DNA sequences for Ta1b (Uniprot Accession # O46167) and Ta1b variants were synthesized by Twist Biosciences (South San Francisco, CA) in a pKlac1 vector and transformed into *Kluyveromyces lactis* (Stell.‐Dekk.) (Van der Walt, 1971) electrocompetent cells prepared as described previously.^13^ Yeast transformants were selected from acetamide nitrogen selection plates and then grown for 4–5 days with shaking at 25°C in 48‐well deep‐well culture plates or shake flasks using a *K. lactis* yeast defined fermentation medium (YDFM).^14^ Cells were pelleted at 10000 *g* for 10 min and the resulting supernatant was collected and passed through a sterile 0.2 μm filter. Expression of peptides was evaluated by HPLC on a Chromolith C18 column (4.6 × 100 mm) using a linear gradient from 15 to 33% acetonitrile with a flow rate of 2 mL min^−1^ over 8 min. The cell‐free media containing the peptide was either used directly in *in vivo* bioassays following concentration with a 3‐kDa MWCO spin filter (Pall Corp., Port Washington, NY) or further purified by ion‐exchange chromatography (IEX) for biochemical studies.

For ion exchange, cell‐free media (2–20 mL) containing peptide samples were pH adjusted to 3.0 with hydrochloric acid and applied to 5 mL SP Sephadex C‐25 (Cytiva, Marlborough, MA) resin equilibrated with 100 mM Glycine buffer pH 3.0 (equilibration buffer). Peptide was applied by gravity flow and washed with 5–10 column volumes (CV) of equilibration buffer followed by 3–5 CV of 30 mM Sodium Acetate, pH 4.5. Peptide was eluted with 3 CV of 30 mM 2‐(N‐morpholino)ethanesulfonic acid, pH 6.0 followed by analysis and quantification by HPLC. N‐terminally truncated mutants required the addition of 200 mM NaCl to the elution buffer to elute. Samples were concentrated with a 3‐kDa MWCO concentrator (Pall Corp.), aliquoted, and stored at −20°C until use.

The Ta1b mutant with four truncated N‐terminal residues (Ta1b‐QA‐ΔEPDE) was ordered as a synthetic peptide (Genscript, Piscataway, NJ). The crude peptide was dissolved in 20 mM Tris–HCl buffer, pH 8.8, and diluted to less than 0.5 mg mL^−1^ and allowed to oxidize slowly by exposure to atmosphere overnight on the benchtop. The folded peptide was then purified by injection on HPLC using the analytical protocol described above and collection of the authentic peak. The sample was dried and rehydrated in 30 mM MES buffer, then quantified, and stored as described above.

### 2.2 Insecticidal activity by injection

Adult house flies (*Musca domestica* L.) were immobilized with CO_2_ gas and those with an average weight of 12–18 mg were selected for injection. Purified solutions of Ta1b and variants were diluted in water and injected into the dorsal thorax with a 1‐cc syringe and 30‐gauge needle using a hand microapplicator (Burkard, Rickmansworth, England). Each adult was injected with 0.5 μL of solution with 10 flies per treatment in at least triplicate. Water was used as a negative control. Flies were placed into a container with moist filter paper and scored for mortality 24 h post‐injection. Ten non‐injected flies were placed in a container to assess CO_2_ mortality. Response curves were fitted with a four‐parameter sigmoidal function (GraphPad Prism, Boston, MA) to determine LD_50_ values.

*Helicoverpa zea* (Boddie, 1850) larvae were reared from eggs (Benzon, Carlisle, PA) to fourth instar on general purpose lepidopteran diet (F9772, Frontier Agricultural Sciences, Newark, DE). Pre‐weighed larvae immobilized on ice were injected into their penultimate proleg as described for *M. domestica* with 1 μL solution and eight larvae per treatment in at least triplicate. After injection, larvae were placed in a well of a 32‐well tray filled with 5 mL lepidopteran diet. The trays were then placed in an incubator at 28° C with 16:8 h light: dark cycle and evaluated 24 h after injection for mortality.

To compare the potency of Ta1b, we also injected the following active ingredients into *M. domestica* and *H. zea*: GS‐ω/κ (Spear, Vestaron), spinosad (Monterey Garden Insect Spray, Monterey), thiamethoxam (Optigard® Flex, Syngenta), and chlorantraniliprole (ALTACOR®, FMC).

### 2.3 Insecticidal activity by drosophila feeding

*Drosophila melanogaster* Meigen, 1830 Oregon‐R wild‐type were starved for 20–24 h, anesthetized using CO_2_, and then 4–7 unsexed individuals were transferred to a well of a 128‐well tray with 1 mL of 1% w/v agar and sealed with a perforated lid. Treatments were prepared by mixing with sucrose to a final concentration of 10% w/v. In a 10 μL pipette tip, 10 μL of treatment was collected in a micropipette tip and the tip was inserted into the lid of the arena. Flies were incubated at 28°C and scored for mortality after 72 h. Treatments were tested in at least triplicate and mortality was calculated using Abbott's formula^15^ followed by extraction of LD_50_ values by fitting to a four‐parameter sigmoidal function (GraphPad Prism, Boston, MA).

### 2.4 Insecticidal activity by foliar spray

Organic romaine lettuce was cut into 30‐mm diameter disks and sterilized with a 140‐ppm bleach solution followed by a triple rinse in water. The lettuce disks were then sprayed on either side using a Potter Tower Sprayer (Burkard, Rickmansworth, England) with insecticidal solutions, allowing them to dry between sprays. Once fully sprayed and dried, disks were placed into an arena, which was a 32‐well rearing tray containing 5 mL of 1% w/v agar. One leaf disk was placed in each well and a single neonate *H. zea* was applied to each of the 12 leaf discs per treatment with four experimental replicates per treatment. The trays were then placed in a 28° C incubator with a 16:8 h light: dark cycle. Mortality was evaluated 4 days after application.

### 2.5 Protease stability assays

Larvae of *H. zea, Spodoptera frugiperda* Smith and Abbot, 1797, *and Trichoplusia ni* Hubner, 1802 were purchased from Benzon Research and reared to fifth instar on general purpose lepidopteran diet. *Manduca sexta* L. were purchased from Carolina Biological Supply (Burlington, NC) and reared to fifth instar on the supplied diet. Larvae were anesthetized on ice for 30 min, pinned on a dissection plate, and opened laterally with microdissection scissors. The foregut, midgut and hindgut were excised and washed using sterile water to remove any excess hemolymph and then transferred into a chilled 2‐mL microcentrifuge tube. The extracts were spun in a microcentrifuge at 10000 *g* to pellet the solids. The liquid fractions were carefully removed, sterile filtered, and stored at −80°C for future use.

For gut enzyme digestion studies, gut extract was diluted 10× with the dilution buffer (30 mM Tris–HCl, pH 8.8) and 0.5–1 mg mL^−1^ of Ta1b. Samples were taken from the digestion mixture at defined timepoints and mixed with a 1:6 ratio of stop buffer (Tris–HCl pH 1–2) to halt further digest of the peptide. Samples were sterile filtered and analyzed by HPLC to determine peptide integrity and concentration.

For commercial enzyme digestion studies, bovine trypsin (Sigma‐Aldrich, St. Louis, MO) was prepared at a concentration of 1 mg mL^−1^ in 50 mM Tris–HCl pH 8.8, 100 mM NaCl. Bovine chymotrypsin (Sigma‐Aldrich) was prepared at a concentration of 1 mg ml^−1^ in 50 mM MES pH 6.0, 100 mM NaCl. For each digestion condition, 5 μL of trypsin or chymotrypsin solution were added to 200 μL of 10.7 mg mL^−1^ peptide and incubated at room temperature. Samples were taken at defined timepoints, sterile filtered, and analyzed immediately by HPLC.

### 2.6 Circular dichroism

Thermal denaturation profiles were collected on a Jasco‐J1500 CD spectrophotometer in a 0.2‐cm cuvette at a peptide concentration of 0.4–0.6 mg mL^−1^ in 10 mM sodium phosphate, pH 7.0. Ellipticity was monitored at 220 nm every 1°C up to 95°C at a rate of 2°C min^−1^. Denaturation curves were fitted with a Boltzmann sigmoidal function (GraphPad Prism) to determine the melting point (*T*_m_) for a single replicate. 3–4 replicates were run and the calculated *T*_m_ values of each variant peptide was tested for a statistically significant difference relative to the rTa1b peptide or Ta1b‐QA commercial peptide, as appropriate, using an unpaired, two‐tailed, heteroscedastic student t's test (Microsoft Excel, Redmond, WA).

### 2.7 Mass spectrometry

Ta1b samples for mass spectrometry (MS) were separated by HPLC on a Chromolith C18 column (4.6 × 100 mm) and a gradient of 10–40% acetonitrile over 20 min to collect Ta1b peaks of interest. Samples were reduced with dithiothreitol at 60°C followed by alkylation with iodoacetamide then treated with trypsin or chymotrypsin at a ratio of 1:10 enzyme‐to‐peptide at 37°C for 18 h. MS and MS/MS were acquired by MS Bioworks (Ann Arbor, MI) on a Q Exactive quadrupole‐orbitrap (Thermo‐Fisher, Waltham, MA) at 60000 FWHM and 15 000 FWHM resolution, respectively. Post‐translational modifications were determined in Scaffold PTM using A‐Score.^16^

### 2.8 Field trials

A dose–response of U1‐AGTX‐Ta1b‐QA (Ta1b‐QA) combined with *Bacillus thuringiensis* subsp. *kurstaki (Btk;* DiPel DF®, Valent) was performed by Agricola 2000 in a randomized complete block design (RCBD) on cabbage caterpillar (*Pieris brassicae* L.) on cabbage in Palagiano, Italy with four replicate plots of 1 × 15 m in open‐field containing 25 plants each. Treatments included an untreated check, three rates (22, 34, 45 g AI ha^−1^) of an 8.5% w/w spray‐dried sample of Ta1b‐QA together with Btk (DiPel DF®, Valent, 0.5 kg product ha^−1^), and the reference standard spinosad (Laser®, Corteva, 96 g product ha^−1^). Treatments were applied two times, 7 days apart, with a backpack CO_2_ sprayer in a spray volume of 800 L ha^−1^. The number of caterpillars per plant was counted 1 days after the second application, and these data were converted to % control relative to the untreated check using Abbott's formula. Data were analyzed by one‐way ANOVA followed by a Tukey HSD LSM test with significance set at *P* < 0.05 (GraphPad Prism).

A rotation of Ta1b‐QA with chemical standards was performed by Bisabri Ag on navel orangeworm (*Amyelois transitella* (Walker, 1863)) on almonds in Newman, *CA*. The study was a randomized complete block design (RCBD), in open‐field, with treatments replicated four times, plot size 6.4 × 8.5 m each with a single tree. Treatments included an untreated check, one rate (47.6 g AI ha^−1^) of an 8.5% w/w spray‐dried sample of Ta1b‐QA together with Btk (Leprotec™, Vestaron, 1.17 L product ha^−1^), and two reference standards methoxyfenozide (Intrepid 2F®, Corteva, 1.74 L product ha^−1^) and chlorantraniliprole (Altacor®, FMC, product 0.33 L product ha^−1^). A non‐ionic surfactant Dyne‐Amic™, Helena Agri‐Enterprises, 0.0625% v/v) was added to all treatments. Treatments were applied two times, at 1–3% almond hull split and then 16 days later at about 80% hull split, with an air‐blast sprayer calibrated to 1169 L ha^−1^. Ta1b‐QA was tested as a self‐rotation (two applications) or rotated with methoxyfenozide as either the first or second application, and also compared to the standard rotation of methoxyfenozide followed by chlorantraniliprole. At 42 days after the first application 250 kernels randomly selected from each plot were assessed for damage, and % damage was analyzed by one‐way ANOVA followed by a Tukey HSD LSM test with significance set at *P* < 0.05 (GraphPad Prism).

Contact activity of Ta1b‐QA on green peach aphids (*Myzus persicae* (Sulzer, 1776)) on cucumbers in the greenhouse was performed in cooperation with A.C.D.S Research in North Rose, NY. The study was a randomized complete block design (RCBD), with treatments replicated six times, plots consisting of three pots each containing two to three plants. Treatments included an untreated check, three rates (1, 2, 3% v/v) of a 16.2% w/w liquid sample of Ta1b‐QA mixed with a non‐ionic surfactant (Activator®, Loveland Products) at 0.125% v/v, and a reference standard imidacloprid (Admire Pro®, Bayer, 0.088 L product ha^−1^). Treatments were applied four times, at 6–8‐day intervals, with a backpack CO_2_ sprayer in a spray volume of 374 L ha^−1^. The number of aphids per plant was counted at regular intervals (0, 4, 11, 17, 23 and 29 d after first application). Cumulative insect‐days (defined as the area under the insect population curve as a measure of pest pressure) were calculated based on those six rating dates, then converted to % control relative to the untreated check using Abbott's formula. One replicate was removed due to a lack of pest pressure in the untreated check. Data were analyzed by one‐way ANOVA followed by a Tukey HSD LSM test with significance set at *P* < 0.05 (GraphPad Prism).

## 3 RESULTS

### 3.1 Recombinant Ta1b shows potent insecticidal activity

**Figure 1:**
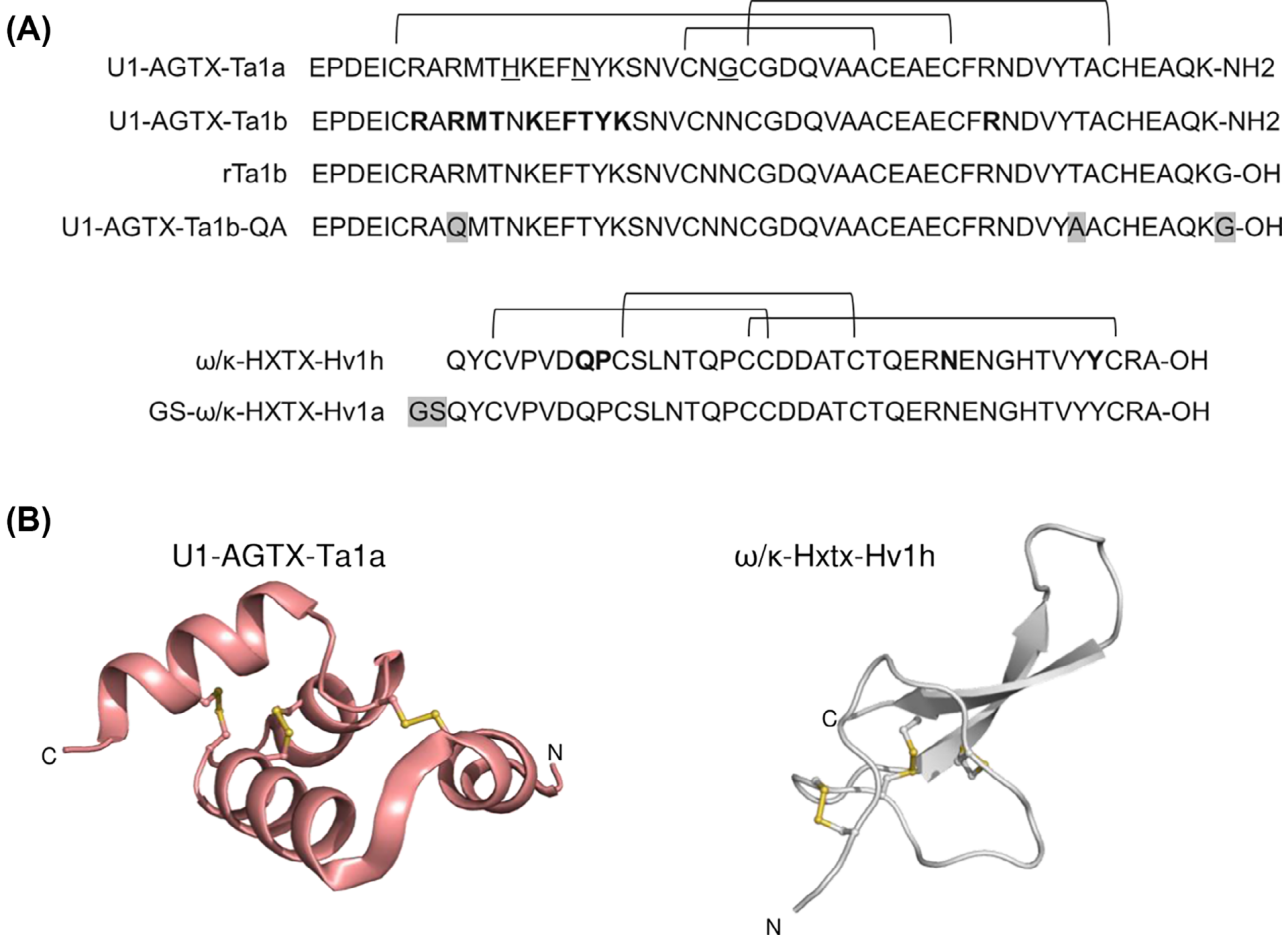
Venom peptide sequence and structure overview. (A) Sequences of note for the Ta1 and Hv1 peptides, respectively. Differences between the commercial peptide sequence and the native are highlighted in grey and critical amino acids for activity are bolded. Differences between Ta1a (Uniprot Accession # O26166) and Ta1b (Uniprot Accession # O46167) are underlined. Disulfide bonds are indicated with connecting brackets above the sequences. (B) Structures of the native peptides from each family. Ta1a was modeled from PDB structure 6URP and Hv1h from structure 2H1Z.

Native U1‐AGTX‐Ta1b is post‐translationally processed in the spider venom gland to remove the C‐terminal glycine followed by amidation of the subsequently terminal lysine residue (Fig. 1A).^17^ It was not possible to produce amidated U1‐AGTX‐Ta1b recombinantly, so rTa1b was produced in yeast with (referred as rTa1b in Table 1) and without the terminal glycine residue (referred as rTa1b ΔG in Table 1) and no difference in activity was observed (Table 1). Additionally, the LD_50_ values for rTa1b were similar to published results for U1‐AGTX‐Ta1b isolated from venom,^17^, ^18^ suggesting amidation does not contribute significantly to activity (Table 1). Therefore, further studies were performed using rTa1b containing the C‐terminal glycine and will be referred to as Ta1b.

**Table 1:** Insecticidal LD_50_ values

Insecticide	Injection, nmol g^−1^ (95% CI)	
	*M. domestica*	*H. zea*
U1‐AGTX‐Ta1b	0.7^a^	1.0^b^
rTa1b ΔG	0.24 (0.22–0.27)	2.7 (2.2–3.3)
rTa1b	0.27 (0.23–0.32)	2.4 (1.7–3.4)
GS‐ω/κ	0.19 (0.17–0.21)	5.9 (4.9–6.9)
Spinosad	1.5 (0.9–2.4)	0.20 (0.14–0.31)
Thiamethoxam	23.2 (18.1–29.6)	31.9 (20.2–52.1)
Chlorantraniliprole	no activity	0.22 (0.15–0.37)

^a^ Johnson *et al*. Archives Insect Biochem. Phys. (1998).

^b^ U.S. Patent 08171383.

### 3.2 Ta1b is not orally active against lepidopterans

Due to their size and physical properties, peptide‐based biologics have difficulty crossing insect external barriers, such as the cuticle and gut epithelium, and often have reduced bioavailability relative to their small‐molecule counterparts.^19^, ^20^, ^21^, ^22^ To address this shortcoming, a commercial formulation of GS‐ω/κ‐HXTX‐Hv1a was developed where it is co‐applied with the gut targeting bioinsecticide derived from *Bacillus thuringiensis* subsp. *kurstaki* (Btk). Strains of *B. thuringiensis* produce crystal protein toxin complexes that bind to target receptors specifically in many lepidoptera insect midgut, causing permeabilization through direct action on epithelial cell membranes.^23^, ^24^ In this model, permeabilization leads to increased bioavailability for the GS‐ω/κ peptide.

**Figure 2:**
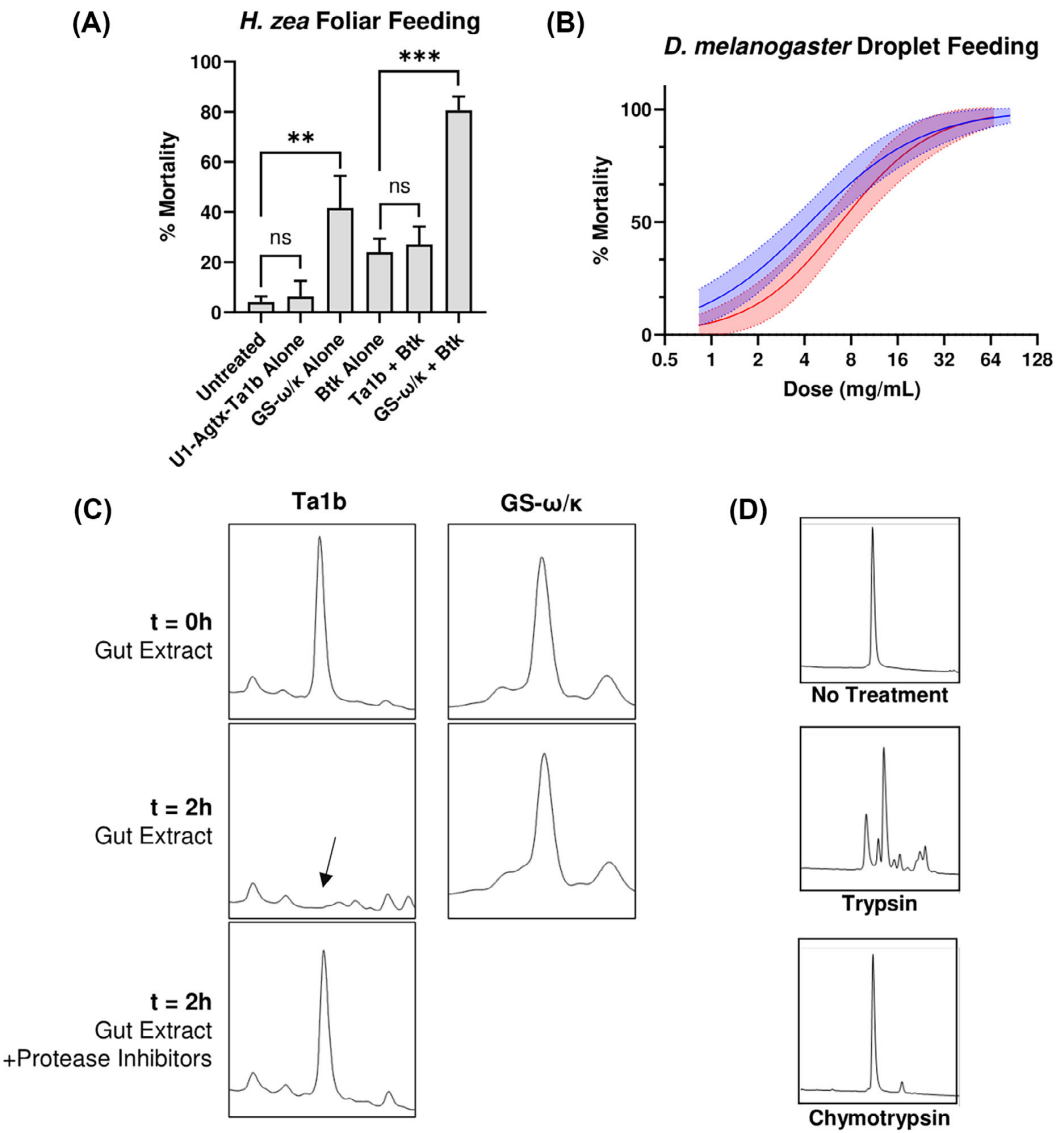
Ta1b is inactivated by gut proteases from Lepidopteran but not Dipteran insects. (A) Day 4 Mortality of *H. zea* on lettuce leaves treated with U1‐AGTX‐Ta1b or GS‐ω/κ (100 μg cm^−2^) either alone or in combination with *Btk* (0.17 μg cm^−2^). Error bars are standard errors of the mean (SEM), and asterisks indicate *P* < 0.01 and *P* < 0.001 for ** and ***, respectively, determined by unpaired Student's *t*‐test. (B) 72 h Mortality of *D. melanogaster* when administered Ta1b (red) or GS‐ω/κ (blue) by droplet feeding. LD_50_'s are 4.4 and 6.9 mg mL^−1^ for GS‐ω/κ and Ta1b, respectively, and were calculated by fit to a four‐parameter sigmoidal function with 95% confidence intervals shaded. (C) HPLC chromatograms of Ta1b (left) or GS‐ω/κ (right) incubated with *Manduca sexta* gut extract. Arrow indicates loss of Ta1b peak upon incubation with gut extract. (D) HPLC chromatograms of Ta1b incubated with bovine trypsin or chymotrypsin.

Based on this commercial formulation, we developed a lab‐based assay for assessing the oral activity of peptide‐based insecticides against lepidopteran pests using sublethal doses of Btk in combination with candidate peptides. GS‐ω/κ had significant activity on its own against *H. zea* neonates compared to untreated, and its activity is increased when co‐applied with a sublethal dose of Btk (Fig. 2A). Unlike GS‐ω/κ, however, Ta1b had no activity on its own or in combination with Btk (Fig. 2A). Thus, despite possessing potent activity by injection, Ta1b is not orally active against *H. zea* larvae.

To determine whether the lack of oral activity was broadly applicable or unique to lepidopterans, we tested GS‐ω/κ and Ta1b by feeding the dipteran insect *D. melanogaster*. Although previous studies indicated a lack of oral toxicity for Ta1a,^18^ Ta1b had similar potency to GS‐ω/κ in fruit flies suggesting the lack of oral activity of Ta1b in *H. zea* was due to a difference in larval lepidopteran digestive tracts from dipterans (Fig. 2B).

### 3.3 Ta1b is degraded by lepidopteran gut proteases

The liquid contents of *M. sexta* gut were extracted and incubated with Ta1b to determine its stability in the presence of lepidopteran gut enzymes. As evidenced by the disappearance of the Ta1b HPLC peak, the protein was rapidly degraded when incubated with gut extract with a half‐life under 2 min (Fig. 2C, Supplemental Fig. 2A). The degradation could be blocked by the addition of EDTA‐free protease inhibitor cocktail, indicating proteases were responsible for the loss of peptide (Fig. 2C). Notably, GS‐ω/κ did not show any degradation when incubated with gut extract, consistent with its oral activity.

Serine proteases, such as trypsin and chymotrypsin, are known to be the predominant protease in lepidopteran guts, although other proteases are also present.^25^, ^26^ To determine whether serine proteases were responsible for Ta1b degradation, we incubated the peptide with bovine trypsin and chymotrypsin. Trypsin, but not chymotrypsin, resulted in digestion of the peptide as indicated by the formation of several new peaks in the HPLC chromatogram (Fig. 2D). In contrast to incubation of peptide with gut extract in which all detectable protein disappeared, trypsin digestion alone resulted in several different peptide fragments that were detected, indicating that additional enzymes likely contribute to Ta1b instability in the native insect gut environment (Fig. 2C,D).

### 3.4 Mutation scan to identify changes that prevent degradation and activity loss

To identify potential mutations that would reduce the susceptibility of Ta1b to degradation by trypsin, we systematically assessed the proteolytic stability of mutants containing alanine substitutions at the five arginine and lysine residues, excluding the C‐terminal lysine Ala mutant. The mutants at three positions showed a marked decrease in degradation rate by bovine trypsin, with one mutant (R9A) showing complete abrogation of Ta1b digestion (Table 2). Two variants, R9A and R38A, were further tested in *M. sexta* gut extract and found to be 213‐ and 9.2‐fold more stable, respectively, indicating trypsin digestion was responsible for the majority of gut instability. Although incubation of Ta1b with bovine trypsin resulted in several degradation products, it appears the single mutation of R9A is sufficient for protection of the protein against degradation in the insect gut.

**Table 2:** Proteolytic stability evaluation of selected Ta1b amino acid alanine scan mutants

Mutant	Degradation half life (min)		Degradation half time ratio to Ta1b	
	Trypsin	Gut extract	Trypsin	Gut extract
Ta1b	288	1.4	‐‐	‐‐
R7A	132	N/D	0.5	N/D
R9A	>6000	298	>20	213
K13A	294	N/D	1.0	N/D
K18A	1194	N/D	4.1	N/D
R38A	1860	12.9	6.5	9.2

*Note*: N/D not determined if susceptive to trypsin.

While the R9A mutant of Ta1b is highly protective against proteolysis by gut enzymes, *M. domestica* injections of R9A resulted in loss of insecticidal activity (Fig. S1). To identify mutations at or near the R9 site that would both prevent proteolytic degradation and preserve bioactivity, we tested several mutations positions before and after R9, avoiding M10 as it was an important pharmacophore residue in the alanine scan. Mutants were first tested for *M. domestica* activity, and those that maintained activity were then subjected to gut extract stability testing (Table 3). Of the 11 mutations screened, three (R9N, R9Q, and T11P) showed stability enhancement up to the limit of testing (50‐fold) with similar bioactivity to Ta1b, with R9Q showing the best combination of activity and stability (Table 3).

**Table 3:** Mutation scan for proteolytically stable and active Ta1b variants

Mutant	Fold stability improvement over Ta1b	Fold activity reduction over Ta1b
A8D	Not tested	>10
A8N	1.8	2.4
A8P	Not tested	>10
A8S	12.1	1.3
R9A	213	8.7
R9E	Not tested	>10
R9H	16.2	2.5
R9N	>50	1.5
R9Q	>50	0.8
T11D	Not tested	>10
T11P	>50	1.4

*Note*: Not tested if mutants showed dramatic activity loss.

To determine if the R9Q mutation of Ta1b conveyed stability against additional agriculturally relevant pests, it was tested against gut extracts from *H. zea, S. frugiperda*, and *T. ni*. Degradation of Ta1b was fitted to a single exponential decay with half‐lives of 0.5–2 h in 10‐fold diluted gut extract, whereas R9Q had too little degradation to calculate half‐lives even after 21 h (Fig. S2B). Therefore, the R9Q mutation is sufficient to broadly protect Ta1b from proteolytic degradation by lepidopteran proteases *in vitro*.

**Figure 3:**
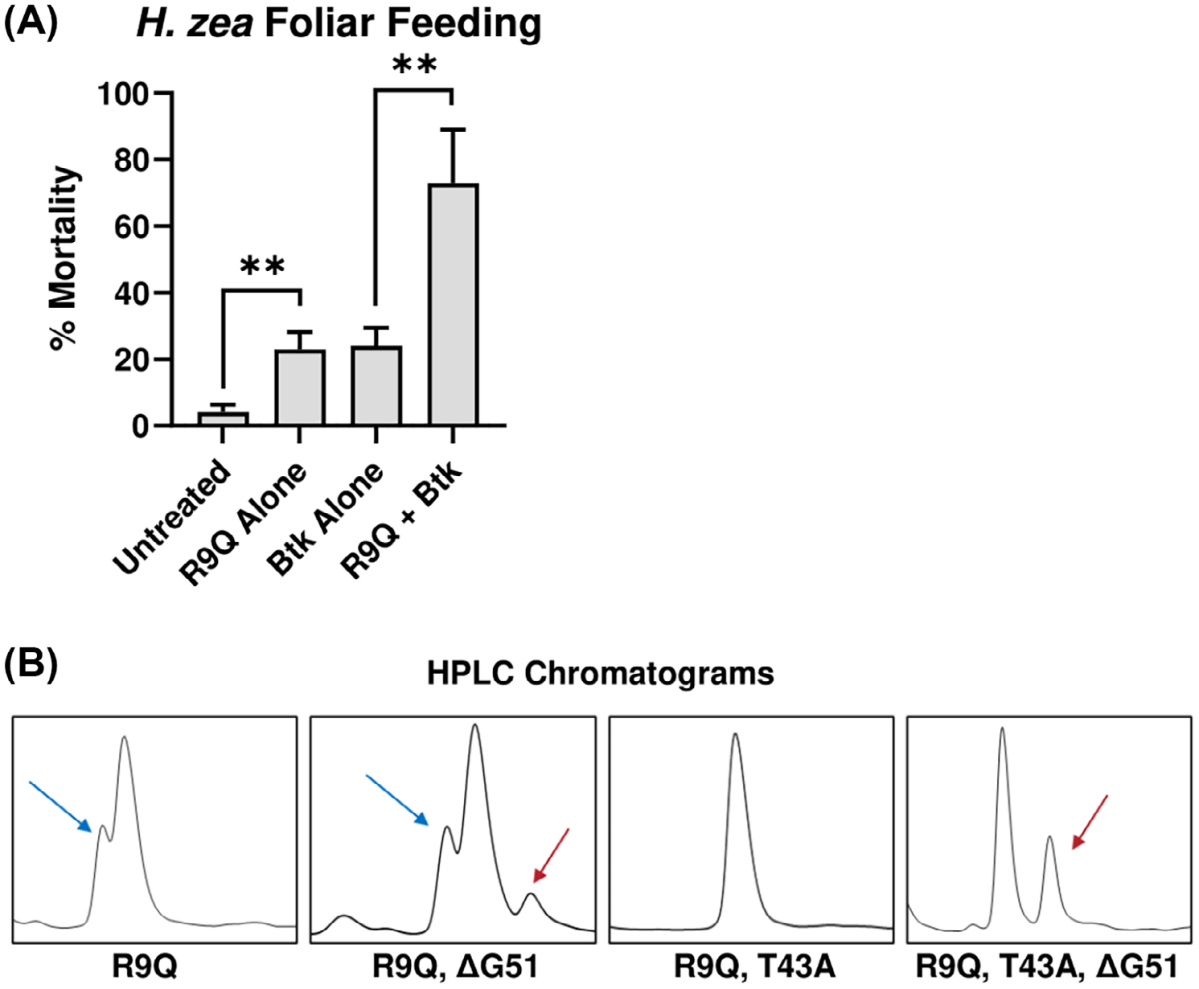
Activity and optimization of Ta1b‐R9Q. (A) Day 4 Mortality of *H. zea* on lettuce leaves treated with Ta1b‐R9Q (100 μg cm^−2^) with and without co‐application of *Btk* (0.17 μg cm^−2^). Error bars are standard errors of the mean (SEM) and asterisks (**) indicate *P* < 0.01 determined by unpaired Student's *t*‐test. (B) Mass spectrometry analysis of the yeast secreted peptide could identify two species that were resolvable in reverse‐phase HPLC with an elongated acetonitrile gradient. One species is a 162 Da mass adduct determined to be a sugar attached to residue T43 (blue arrows). This species disappears upon mutation of T43 to an alanine. The second is loss of lysine 50 when glycine 51 is not present (red arrow).

To determine whether the R9Q mutation could make Ta1b active by oral ingestion, we again performed the foliar feeding assay with *H. zea* neonates. Unlike tests with wildtype Ta1b, the R9Q mutant was significantly insecticidal both alone and in combination with *Btk* (Fig. 3A). To the best of our knowledge, this is the first published incidence of protease stabilization of a venom peptide granting oral activity in an insect. This strategy could have significant implications for improving oral bioavailability for venom peptides to be used as insecticides.

### 3.5 Generation of final peptide sequence for commercialization

A single, stable peptide sequence is critical for regulatory and commercial success of an insecticide. As indicated by the multiple peaks present on the HPLC chromatograph, yeast‐produced Ta1b‐R9Q was not a single species (Fig. 3B). Mass spectrometry analysis identified a 162 Da mass shift present on a fraction of the total peptide, consistent with the addition of a sugar moiety. We were able to localize the mass shift to position T43 using tandem MS/MS, suggesting the adduct was a O‐linked mannosylation, a glycosylation commonly found in the cell wall and secreted proteins in budding yeast.^27^, ^28^ With the mutation T43A, we were able to prevent mannosylation without apparent loss in bioactivity (Fig. S4). Additionally, removal of the C‐terminal glycine to more closely match the mature spider peptide resulted in a small fraction of peptide where the C‐terminal lysine residue was removed. Lysine removal was most likely the result of a yeast exoprotease acting during the secretion process since we could not detect additional loss of the lysine residue upon incubation in cell‐free media over 1–2 weeks (data not shown). However, as we previously established that the C‐terminal glycine has no impact on activity (Table 1), Ta1b‐R9Q‐T43A was designed to include the terminal glycine to prevent the loss of the penultimate lysine, denoted as Ta1b‐QA (see Fig. 1A for full sequence).

### 3.6 Ta1b alanine scan identification of pharmacophore

**Figure 4:**
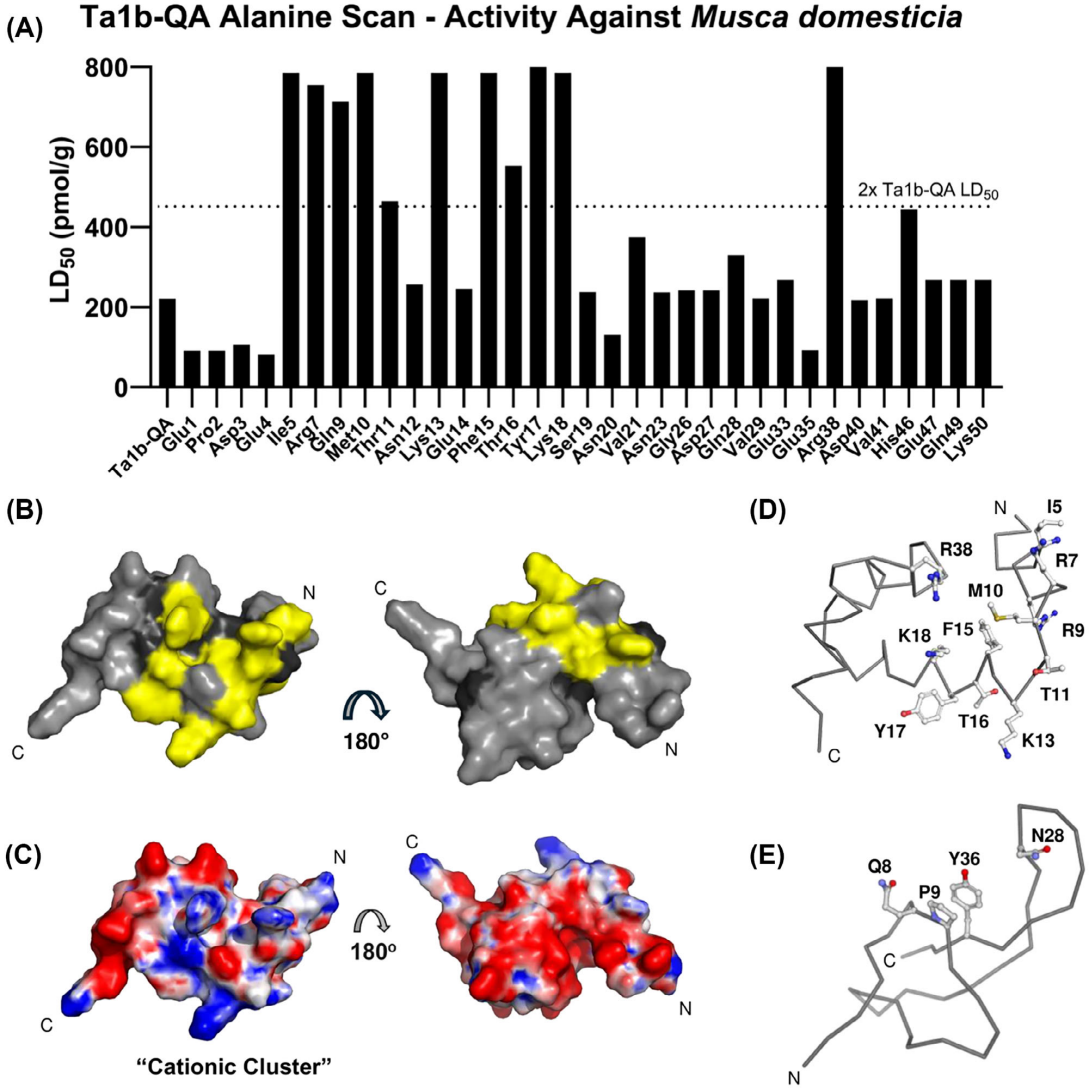
Alanine Scan and Pharmacophore Analysis of Ta1b‐QA. (A) Alanine scan on Ta1b‐QA shown as the estimated concentration for 50% mortality (LD_50_) with amino acid and position along x‐axis. All mutants were tested by injection in adult *M. domestica* with peptide dilutions ranging in concentration from 30 to 800 pmol g^−1^ (up to ≈4× the LD_50_ of Ta1b‐QA). The mutated residues showing 2× or more reduction in potency were determined to be critical for activity. Positions N24, F37, N39, Y42 were not tested due to lack of expression and the terminal position G51 was not tested as it was already determined to be unnecessary for activity. (B) Surface view of sites critical for activity (yellow) for Ta1b overlayed on Ta1a structure (PDB 6URP). Sites that correspond to cysteine, alanine, or where the alanine mutation disrupted peptide expression are shaded in dark grey. (C) Solvent accessible surface electrostatics of Ta1a were calculated at pH 7.0 using Adaptive Poisson‐Boltzmann Solver (APBS). Surface is modeled from −5 kT/e – +5 kT/e with negative charges modeled as red, neutral as white, and positive as blue. (D) Critical amino acids displayed on the peptide backbone comparing Ta1b and (E) ω/κ‐Hv1h (PDB 2H1Z).

To identify the key residues for activity, an alanine scan was conducted for all non‐alanine or cysteine residues and then activity was assessed *via* injection at a single high dose into adult *M. domestica*. All but five constructs, N24A, F37A, N39A, Y42A and G51A, were successfully expressed and evaluated for bioactivity by adult *M. domestica* injections. Results indicate a linear sequence stretching from position 5–18 that is critical for bioactivity (Fig. 4A). These residues cluster, along with R38, on the structure to present the presumed binding interface with the peptide's target receptor as the yellow stretch on the peptide surface shown in Fig. 4B, and they present strong positively charged peptide surface (Fig. 4C). This cluster contains five basic amino acids (R7, R9, K13, K18, R38), that we term the ‘cationic cluster’, suggesting ionic interactions are critical for bioactivity. In fact, dose–response curves were generated for R9A, K13A, and K18A that showed 6.6‐, 13.4‐, and > 60‐fold losses in activity, respectively, confirming their biological importance (Fig. S1). To our knowledge, this is the first alanine scan of a HAND peptide toxin, and the putative pharmacophore differs substantially from that of ω/κ‐Hv1h^24^ which does not possess any basic amino acids essential for its insecticidal activity (Fig. 4D).

### 3.7 Ta1b‐QA has high thermal stability due to N‐terminal residues

Consistent with the high stability reported for other cysteine‐rich venom peptides,^29^ Ta1b possesses a high melting temperature (*T*_m_ = 73.7 ± 0.4) that is notably higher than the previously reported melting temperature for the closely related toxin Ta1a (Tm = 51.0 ± 0.4).^30^ The R9Q mutation caused a slight, but not statistically significant, increase to the melting temperature (*T*_m_ = 76.5 ± 0.8), while the double mutant with T43A (Ta1b‐QA) had no additional impact (T_m_ = 75.1 ± 0.3, Fig. S3 and Table S1). Ta1b and variants did not show visible precipitation during the heating step, but reached only a fraction of their previous level of α‐helical content after cooling back down, indicating partial refolding on the timescale of the experiment (≈2 h, data not shown).

Based on the alanine scan screen, the first four N‐terminal amino acids may be dispensable for activity, so systematic truncations of the N‐termini were analyzed. N‐terminal truncations had no or little impact on bioactivity by adult *M. domestica* injection bioassays (Table S1), but thermal stability of Ta1b‐QA decreased significantly with each additional residue removal, culminating with a 14.2°C reduction in melting temperature for the mutant incorporating the deletion of residues E‐P‐D‐E from the N‐terminus (Fig. S3 and Table S1). Thus, while the acidic N‐terminus does not appear essential for bioactivity, it confers substantial stability to the overall structure and fold of Ta1b‐QA.

### 3.8 Ta1b‐QA is an orally active biopesticide in field trials

**Figure 5:**
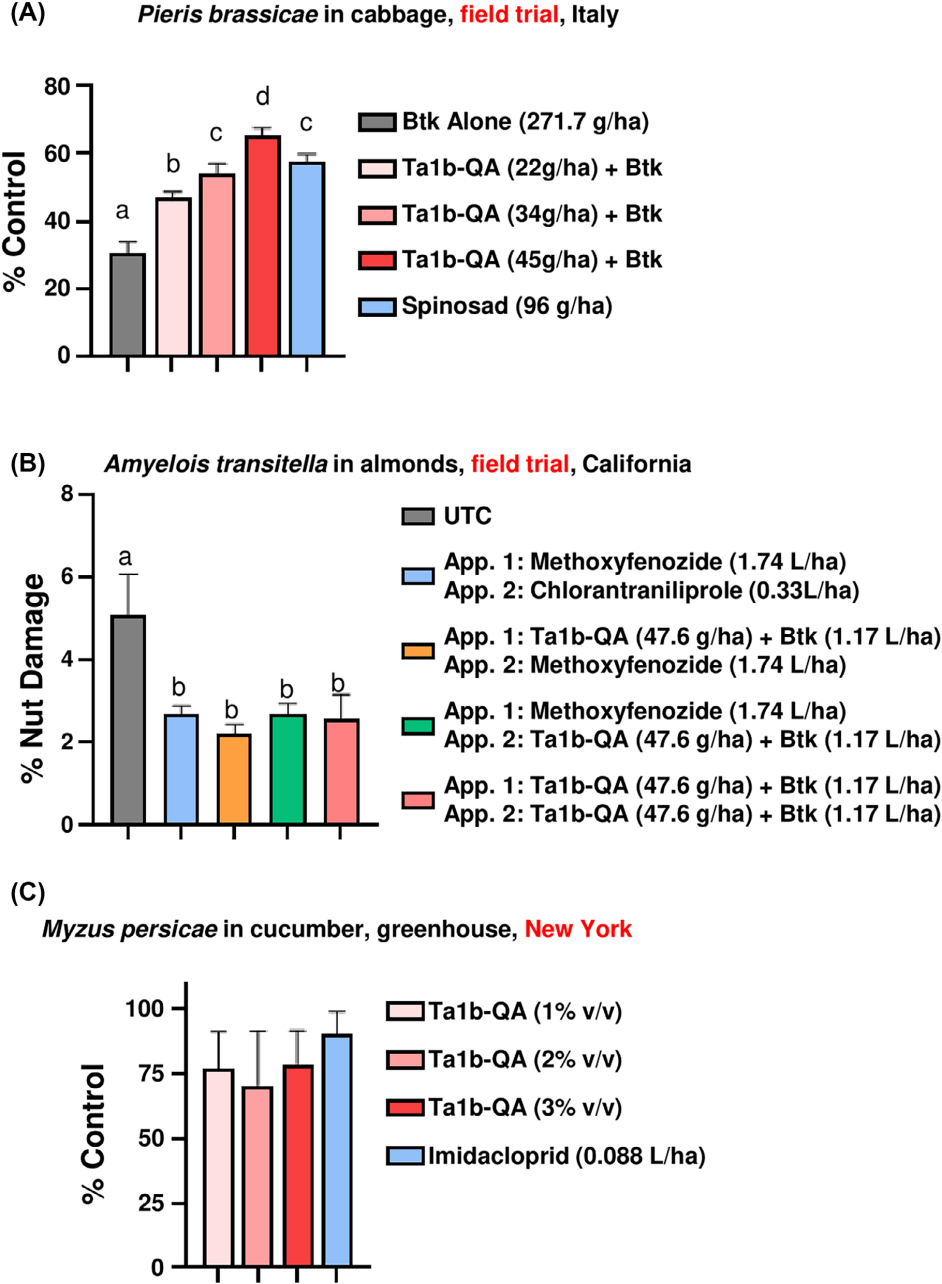
Field trials. (A) Replicated field trial of Ta1b‐QA applied in combination with *Btk* against *Pieris brassicae* on cabbage compared to a conventional standard applied at the manufacturer's recommended rate. Data are % control of larvae relative to the untreated check. (B) Replicated field trial of Ta1b‐QA in combination with *Btk* against *Amyleois transitella* on almonds. A self‐rotation of Ta1b‐QA + *Btk* was compared to a conventional rotation in which one product (chlorantraniliprole) was substituted for Ta1b‐QA + *Btk*. UTC = untreated check. (C) Greenhouse trial of Ta1b‐QA against *Myzus persicae* on cucumber compared to imidacloprid at the manufacturer's recommended label rates. Data are % control of cumulative insect‐days relative to the untreated check. Error bars are standard errors of the mean (SEM).

In the field trial against *P. brassicae* in cabbage there was a significant effect of treatment, with all treatments demonstrating efficacy against larvae (Fig. 5A). Mean % control of larvae relative to the untreated check was 47.2, 54.1 and 65.3% across the three doses of Ta1b‐QA with *Btk*, including a significant positive dose response. The middle dose was statistically equivalent to the reference standard spinosad (57.8%), while the highest dose was significantly greater. All three doses were significantly greater than *Btk* alone (30.6%). These results confirm that Ta1b‐QA in a tank mix with *Btk* outperforms *Btk* alone and can perform as well as conventional standards.

In the field trial against *A. transitella* in almond there was a significant effect of treatment but no difference among treatments (Fig. 5B). Compared to the untreated check (7.0% damage), two applications of Ta1b‐QA (3.3% damage), or one application rotated before (2.7%) or after methoxyfenozide (4.2%) showed performance equivalent to the standard rotation (3.6%) (Fig. 5B). These results confirm that a two‐application program with Ta1b‐QA can perform as well as a conventional program, and also that Ta1b‐QA can be incorporated into a rotation program alongside conventional products as a new mode of action to reduce the risk of insecticide resistance development.

In the field trial against *M. persicae* in greenhouse cucumber there was a significant effect of treatment but no difference among treatments (Fig. 5C). Mean % control of cumulative insect‐days relative to the untreated check was 77.3, 70.5 and 79.0 across the three doses of Ta1b‐QA. All three doses were statistically equivalent to the reference standard imidacloprid (90.4% control). These results confirm that against small, softer‐bodied insects like aphids, and also mites, thrips and whiteflies (unpublished data) Ta1b‐QA has contact activity.

## 4 DISCUSSION

U1‐AGTX‐Ta1b was isolated from the venom of the hobo spider in the late 1990's and was noted to possess strong insecticidal activity upon injection into a wide range of insect pests, particularly those in the order Lepidoptera, while possessing no toxicity towards mammals.^17^, ^31^ We confirmed that the Ta1b peptide can paralyze and kill insects with potencies similar to other widely available insecticides, demonstrating its potential as a bioinsecticide lead. When injected into *M. domestica* adults and *H. zea* larvae, both Ta1b and GS‐ω/κ show strong insecticidal activity (Table 1) that compare favorably in potency to several central nervous system (CNS) acting small‐molecule insecticides (Table 1), demonstrating the potential of Ta1b as a bioinsecticide candidate.

Ta1b shares some structural similarities to GS‐ω/κ, another peptide‐based insecticide. Both toxins are relatively small and stabilized with three disulfides; however, their overall 3‐dimensional structure is greatly divergent (Fig. 1B). The structure for Ta1b is defined as a helical arthropod‐neuropeptide‐derived toxin (HAND) and is comprised of four alpha helices locked together with three disulfide bonds^30^; whereas, GS‐ω/κ, and its parent molecule ω/κ‐Hv1h, have an inhibitor cystine knot fold which is defined by its antiparallel β‐sheet loop being stabilized by three disulfides.^32^ The disulfide rich fold and architecture has been shown to protect some venom peptides from proteolytic digestion.^29^ Ta1b however, lacked oral activity against *H. zea* due to rapid degradation in the caterpillar digestive system, yet possessed oral activity against *D. melanogaster* adults in line with the commercial peptide insecticide GS‐ω/κ.

Proteases in the insect gut are important for breaking down ingested proteins as well as for defense against microbial or viral infection.^33^, ^34^ While some protein based insecticides, like Cry1Ac,^35^ are activated by proteases found in the insect midgut, the proteases are likely also responsible for inactivating other protein based toxins when fed.^18^, ^21^, ^36^ Thus, we hypothesized that Ta1b may be susceptible to degradation by proteases in the lepidopteran gut, leading to its loss of efficacy in oral presentations. Digestion with the commercially available proteases revealed that a trypsin‐like serine protease was likely to be responsible for inactivating Ta1b in an oral presentation to *H. zea*, consistent with the observation that serine proteases are highly abundant in lepidopteran digestive tracts.^25^, ^26^ Interestingly, despite possessing six basic amino acids that could theoretically be sites for proteolysis by trypsin‐like enzymes, a single point mutation at position R9 sufficiently stabilized the peptide against cleavage and rendered the peptide orally bioactive when changed to glutamine. Although previous studies have suggested that gut stabilization could have an impact on toxicity,^21^, ^36^ our work is the first study to successfully demonstrate a venom peptide made orally efficacious by stabilizing it against insect gut proteases.

The optimized Ta1b sequence contained a removal of a glycosylation site (T43A) in addition to the protease stabilizing mutation (R9Q). This peptide, termed Ta1b‐QA, was tested in replicated field trials to determine its potential as a commercial insecticide. Against lepidopteran targets, a positive rate response was observed with the addition of *Btk* to Ta1b‐QA, with the highest rate outperforming the conventional control. More importantly for growers that must rotate insecticidal chemistries throughout a growing season, Ta1b‐QA was able to substitute for chlorantraniliprole in a rotation with methoxyfenozide. Therefore, Ta1b‐QA could be a useful part of growers' IPM strategies with the added benefit of reducing the use of small‐molecule chemistries while also aiding management of insecticide resistance development.

The mode of action of Ta1b is unknown, but the Ta1 family has been shown to paralyze insects *via* stimulation of ion channels in the central nervous system (CNS),^17^, ^37^ targets that were previously thought to be inaccessible for venom peptides. The spider venom insecticide GS‐ω/κ has recently been shown to be a positive allosteric modulator of nicotinic acetylcholine receptors (nAChR),^38^, ^39^ indicating CNS targeting spider peptides are prevalent and are promising insecticidal agents. A recent study evaluating the nicotinic activity of GS‐ω/κ and Ta1b in *D. melanogaster* nerve cords showed Ta1b activity was surprisingly stimulated by both the nAChR agonist nicotine and antagonist alpha‐bungarotoxin.^37^ GS‐ω/κ activity was stimulated by nicotine and blocked by alpha‐bungarotoxin, as expected for a positive allosteric modulation of nAChR. This phenomenon was also observed in combination studies in *M. domestica* where alpha‐bungarotoxin increased Ta1b activity but blocked GS‐ω/κ activity. Thus, it appears Ta1b interacts with the insect cholinergic system in a yet undefined manner. Further studies will be necessary to determine the mode of action and assess the risk of cross‐resistance for IPM strategies.

In 2024, U1‐AGTX‐Ta1b‐QA was approved as an insecticide in the U.S. and Mexico under the brand name BASIN™ and is currently under regulatory review in additional countries. New peptide bioinsecticides like Ta1b‐QA offer useful tools for growers to maximize crop yields while improving environmental sustainability.

## 5 CONCLUSION

Stabilization of a spider venom peptide to digestive enzymes results in an orally active bioinsecticide. Further optimization of the peptide sequence generated a commercially viable bioinsecticide that has received regulatory approval to control insect pests. It is likely that there exists many more potential bioinsecticide lead compounds from animal venoms that would require similar protease stabilizations to become active by ingestion.

## CONFLICT OF INTEREST STATEMENT

BRD, AMH, JST, DLH, REC, BTD, CJF, LMB, DCP, TTN, LB, RMK, and KDS were employees and/or shareholders of Vestaron Corporation at the time work was performed.

## Supporting information


**Data S1.** Supporting Information.

## Data Availability

The data that support the findings of this study are available from the corresponding author upon reasonable request.
