# Supplementary material for: Proteolytic stabilization of a spider venom peptide results in an orally active bioinsecticide
Source: Pest Manag Sci. 2025 Jun 18;81(10):6404–15. doi: 10.1002/ps.8980 (PMC12441774; doi:10.1002/ps.8980)
Supplement: Supplementary file 1 — Data S1. Supporting Information. [file PS-81-6404-s001.docx]

**Supporting Information**

| **Supplementary Table 1. Melting temperature and activities of Ta1b variant peptides** | | | |
| --- | --- | --- | --- |
|  | **T_m_ (^o^C ± SEM)** | **Housefly LD_50_, pmol/g (95%CI)** | ***H. zea* LD_50_, pmol/g (95%CI)** |
| rTa1b | 73.7 ± 0.4 | 0.27 (0.23-0.32) | 2.4 (1.7-3.4) |
| R9Q | 76.5 ± 0.8 | 0.29 (0.27-0.31) | 2.6 (1.8-3.8) |
| R9Q, T43A | 75.1 ± 0.3 | 0.30 (0.27-0.34) | 2.5 (2.1-3.2) |
| ΔEP | 70.4 ± 0.6 * | 0.40 (0.36-0.46) | n.d. |
| ΔEPD | 64.5 ± 0.1 ** | 0.33 (0.31-0.35) | n.d. |
| ΔEPDE | 60.9 ± 0.4 ** | 0.34 (0.31-0.37) | n.d. |
| n.d. not determined, * p > 0.05, ** p > .001 | |  |  |

**Supplementary Figure 1.** R9A, K13A, and K18A house fly injection.

Dose-response curves for R9A, K13A and K18A were generated by house fly injection.

**Supplementary Figure 2**. Gut stability of trypsin-stabilized mutants.

(A) Mutants R9A and R38A were assessed for stability in *M. sexta* gut extract over time. Time courses were fit to a single exponential decay with calculated half-lives of 1.4, 298, and 12.9 min for Ta1b, R9A, and R38A, respectively. (B) R9Q protects against degradation in additional lepidopteran species. Ta1b degradation was fit to a single exponential decay (black lines) with calculated half-lives of 118.3, 26.4, 38.1 min in *H. zea, S. frugiperda,* and *T. ni*, respectively. R9Q mutants could not be fit to an exponential decay (red lines). Extracts were diluted ten-fold to capture degradation time-course.

**Supplementary Figure 3.** Circular dichroism melt curves for several mutants and N-terminal truncations.

Original ellipticity data at 220 nM for 3 to 4 replicate melts were fit to a Boltzmann sigmoidal curve to generate alpha helical secondary structure melting temperatures as shown below the figure. For visual comparison, each dataset was scaled from the bottom to the top of its curve fit and graphed on the same axes.

**Supplementary Figure 4.** Removal of glycosylation**.**

Mutation of T43A shows no change in activity against house flies (LD_50_ = 310 (Ta1b) and 305 (R9Q T43A) pmol g^-1^) or *H. zea* (LD_50_ = 2560 (Ta1b) and 2550 (R9Q T43A) pmol g^-1^).

Supplementary Figure 1.

Supplementary Figure 2.

**B**

**A**


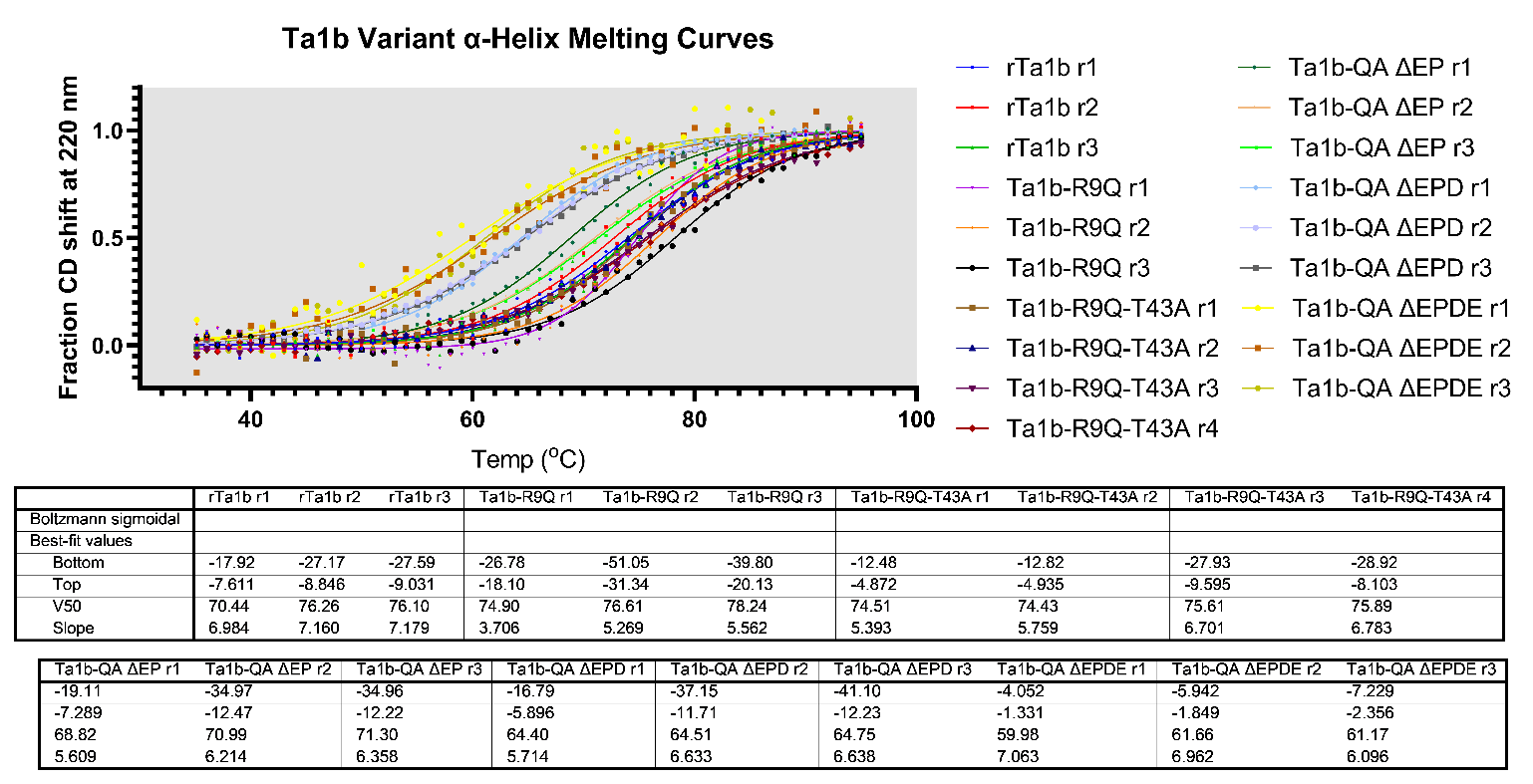
Supplementary Figure 3.

Supplementary Figure 4.

**Dose (pmol/g)**
